# Renal Transplant in a Bardet–Biedl Syndrome Patient: A First Case From Azerbaijan

**DOI:** 10.1155/carm/1963624

**Published:** 2025-10-13

**Authors:** Rashad Sholan, Rufat Aliyev, Nargiz Bakhshaliyeva, Anar Almazkhanli, Rahman Ismayilov, Malahat Sultan

**Affiliations:** ^1^Department of Kidney Diseases and Organ Transplantation, State Security Service Military Hospital, Baku, Azerbaijan; ^2^Scientific Research Center, Azerbaijan Medical University, Baku, Azerbaijan; ^3^Department of Radiology, Azerbaijan Medical University, Baku, Azerbaijan

**Keywords:** Bardet–Biedl syndrome, end-stage renal disease, kidney transplantation

## Abstract

**Introduction:**

Bardet–Biedl syndrome (BBS) is a rare autosomal recessive ciliopathy characterized by multisystem involvement, with renal pathology, including end-stage renal disease, being a major cause of morbidity and mortality.

**Case Presentation:**

This case report presents a 14-year-old boy with BBS and end-stage renal disease who underwent a successful living donor kidney transplantation, marking the first reported case in Azerbaijan. Despite multiple comorbidities, including obesity, hypertension, and high panel reactive antibody levels, the patient achieved stable graft function with a rapid postoperative creatinine decline. Postoperative management focused on immunosuppression and lifestyle adjustments.

**Conclusion:**

This case contributes to the limited literature on BBS, highlighting both the challenges and favorable outcomes of renal transplantation in patients with this rare genetic disorder.

**Summary**

• This case report highlights the successful living donor kidney transplantation in a Bardet–Biedl syndrome (BBS) patient, addressing the unique perioperative challenges associated with obesity, hypertension, and high immunological risk.
• Despite these complexities, careful preoperative planning and a multidisciplinary approach ensured favorable short-term graft function and clinical outcomes.
• This study underscores that renal transplantation is a viable and effective treatment for ESRD in BBS patients, emphasizing the importance of tailored management strategies to optimize long-term success.

## 1. Introduction

Bardet–Biedl syndrome (BBS) is a rare autosomal recessive ciliopathy characterized by multisystem involvement, including renal, ocular, and metabolic abnormalities. To date, at least 26 genes have been identified as causative for BBS, reflecting its considerable genetic heterogeneity [1]. The syndrome presents with hallmark features such as renal anomalies, retinal dystrophy, polydactyly, obesity, hypogonadism, and cognitive impairment [2]. Renal pathology is a leading cause of morbidity and mortality in BBS, with chronic kidney disease (CKD) and, in severe cases, end-stage renal disease (ESRD) affecting a significant proportion of patients [3].

Renal transplantation is the definitive treatment for ESRD in BBS patients. However, it presents unique challenges due to the high prevalence of metabolic syndrome, encompassing obesity, hypertension, hyperlipidemia, and diabetes mellitus in this population. Despite these challenges, transplant outcomes suggest that with proper management, renal transplantation offers favorable long-term survival and graft function for BBS patients [4].

In the literature, only a few case reports and studies address renal transplantation in BBS, underscoring the rarity of such cases and the need for further data to improve patient outcomes [4–7]. This case report details the first documented living donor kidney transplantation for a patient with BBS in the Republic of Azerbaijan, contributing to the body of literature on managing ESRD in BBS.

## 2. Case Report

**Figure 1:**
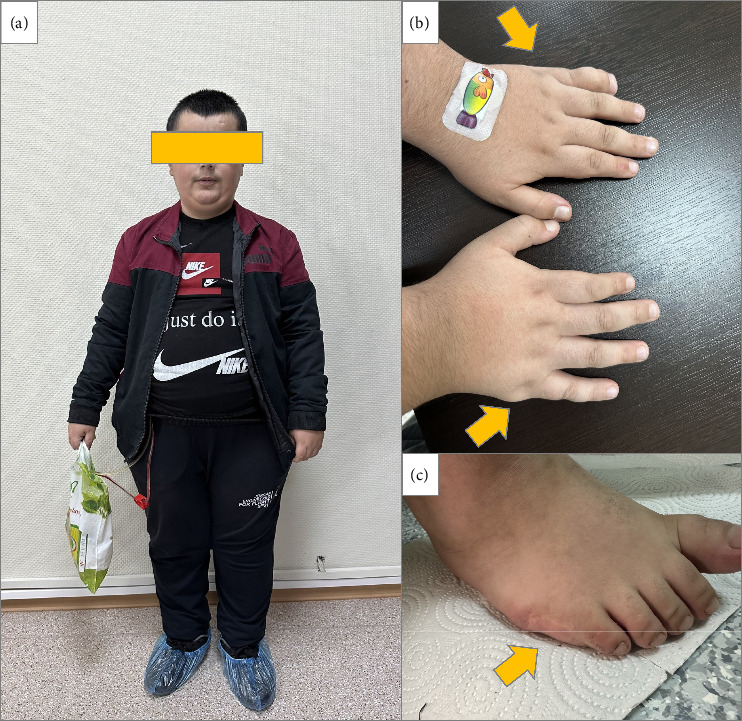
Characteristic phenotypic features in a patient with Bardet–Biedl syndrome: (a) short stature, obesity, and corrective glasses for visual impairment; (b) brachydactyly and residual marking from an amputated extra finger (yellow arrow); (c) close-up view highlighting the amputated extra finger (yellow arrow).

A 14-year-old boy with genetically confirmed BBS was referred to our center for ESRD. Genetic analysis identified a homozygous pathogenic variant in the BBS10 gene (c.271_272del; p.Leu91fs), classified as pathogenic according to ACMG criteria (PVS1, PM2, and PP4). The patient's height was 152 cm and weight 88 kg, corresponding to a BMI of 38.0 kg/m^2^ and a BMI z-score of +3.21 (WHO growth reference). He had a history of obesity since childhood, along with several hallmark features of BBS, including vision impairment due to retinopathy, hypogenitalism (micropenis and small testes), mild developmental delay with learning difficulties, and polydactyly (Figure 1). Renal ultrasonography demonstrated bilateral dysplastic kidneys with loss of corticomedullary differentiation, without cysts or marked hypoplasia. Renal function decline had been progressive since early childhood, with no neonatal renal failure documented. He had been receiving hemodialysis for 4 months prior to referral. Preoperative laboratory results indicated elevated levels of uremic toxins, with creatinine at 6.89 mg/dL (eGFR 8 mL/min/1.73 m^2^, Schwartz formula), blood urea nitrogen at 74 mg/dL, uric acid at 6.9 mg/dL, and parathyroid hormone at 600 mg/dL. Cardiovascular assessment revealed first-degree mitral insufficiency and a third-degree aVF-qR pattern on electrocardiogram, with arterial blood pressure measured at 160/100 mmHg. A psychiatric evaluation identified mild mental deficiency, but no acute psychiatric contraindications to surgery were found.

Due to worsening tolerance of hemodialysis, persistent oliguria, and recurrent catheter-related infections, kidney transplantation was indicated. The donor was the patient's healthy 43-year-old father, with a human leukocyte antigen compatibility of 3/6, panel reactive antibody (PRA) class I at 76%, PRA class II at 89%, and lymphocyte crossmatch results showing T crossmatch at 1.5% and B crossmatch at 89%.

A multidisciplinary team evaluated the patient for transplantation eligibility. Prior to surgery, the patient received a tailored preoperative protocol, which included antithymocyte globulin, steroids, antiviral, antibacterial, and nonsteroidal anti-inflammatory agents adjusted to his weight. The kidney transplantation surgery lasted 3 hours. The second warm ischemia time—defined as the interval from removal of the graft from cold storage to reperfusion—was 38 s, achieved by completing the vascular anastomoses under cold perfusion; the total anastomosis time was 19 min. Cold ischemia time was 1 h and 40 min. A double-J ureteral stent was placed intraoperatively.

The patient exhibited immediate polyuria postoperatively, and creatinine decreased to 3.2 mg/dL on the first postoperative day and stabilized at 1.04 mg/dL (eGFR 92 mL/min/1.73 m^2^) by the second day. The patient was discharged on the sixth postoperative day with comprehensive instructions, including lifestyle modifications and medication guidelines. Immunosuppressive therapy consisted of mycophenolate mofetil, tacrolimus (target trough 8–10 ng/mL), and steroids. Prophylaxis included fluconazole (100 mg/day) and trimethoprim-sulfamethoxazole (400/80 mg/day) for six months. At 2 months postoperatively, kidney function remained stable, with a renal resistive index between 0.69 and 0.72 on Doppler ultrasound, indicating normal graft perfusion.

## 3. Discussion

Structural renal and genital anomalies affect approximately 50% of patients with BBS, making renal and pelvic ultrasonography essential at diagnosis. Annual monitoring of kidney function is also advised, as CKD, a primary cause of early mortality in BBS, may manifest in childhood [8]. The most recent Clinical Registry Investigating BBS (CRIBBS) registry analysis, which evaluated 607 individuals with BBS, identified kidney failure in 44 patients (7.2%), with a median onset age of 12.5 years and the majority of cases occurring before age 30 [9].

Data on renal transplantation in BBS patients are largely derived from case reports [6, 7]. A 2007 study by Sharifian et al. [5] demonstrated that renal transplantation is a safe and effective renal replacement therapy for BBS patients with ESRD, based on findings from five children with a mean age of 11 years (range: 6–17). This study emphasized the importance of monitoring BMI and suggested considering steroid-free immunosuppression, with alternatives like sirolimus and basiliximab, where feasible, to optimize outcomes. Additionally, other case reports highlight specific perioperative concerns for BBS patients, including the risks of airway obstruction and hypothermia during anesthesia, necessitating tailored surgical management [10, 11]. The CRIBBS registry provides the most comprehensive data on renal transplant outcomes for BBS patients to date [4]. Among the 206 enrolled patients, 21 children (16 girls and 5 boys) had developed ESRD, and 18 of these individuals underwent renal transplantation. Graft survival rates were 81.6% at 1 year posttransplant, 75.7% at 5 years, and 49.2% at 25 years. With a median follow-up of 97 months, only a limited number of transplant-related complications were reported, underscoring favorable long-term outcomes. However, BMI was notably elevated in transplanted BBS patients compared to nontransplanted counterparts at the most recent follow-up, highlighting a heightened risk of obesity and metabolic syndrome, which requires vigilant management in this population. Despite these metabolic challenges, the positive long-term outcomes affirm that renal transplantation remains a viable and effective treatment option for BBS patients with ESRD.

In recent years, novel pharmacologic therapies have emerged for managing obesity in BBS, with encouraging clinical results. Setmelanotide, a melanocortin-4 receptor (MC4R) agonist, has demonstrated significant and sustained reductions in body weight and hunger in patients with BBS in a multicenter, randomized, double-blind, placebo-controlled phase 3 trial, followed by an open-label extension [12]. Beyond weight reduction, these benefits may translate into improved metabolic profiles and reduced obesity-related complications, which are of particular relevance in the peri- and posttransplant setting. Furthermore, qualitative research has highlighted positive patient and caregiver experiences with setmelanotide, facilitated by structured patient-support programs [13]. Given the high prevalence of obesity and its metabolic consequences in BBS, incorporating targeted antiobesity pharmacotherapy before and after transplantation may improve not only general health outcomes but also long-term graft survival and cardiovascular risk in this population.

Our case represents the first documented living donor kidney transplantation for a BBS patient in Azerbaijan, adding to the limited literature on transplant outcomes for this rare syndrome. The patient's multiple comorbidities, such as obesity, hypertension, and elevated PRA, underscore the unique perioperative and postoperative challenges. The patient tolerated the procedure well, with a rapid decrease in creatinine levels postsurgery and no immediate postoperative complications. Despite the patient receiving a steroid-based immunosuppressive regimen, his weight remained stable due to strict adherence to dietary and lifestyle recommendations, with even a slight decrease observed. By the second postoperative month, graft function remained stable, and the steroid-inclusive immunosuppressive regimen was continued, given the high PRA and associated rejection risk.

## Data Availability

Data supporting this case report are available from the corresponding author upon reasonable request.
